# Perioperative anesthesia care for the pediatric patient undergoing a kidney transplantation: An educational review

**DOI:** 10.1111/pan.14271

**Published:** 2021-08-20

**Authors:** Marieke Voet, Elisabeth A. M. Cornelissen, Michel F. P. van der Jagt, Joris Lemson, Ignacio Malagon

**Affiliations:** ^1^ Department of Anesthesiology, Pain and Palliative Medicine Radboud University Medical Center Nijmegen the Netherlands; ^2^ Department of Pediatric Nephrology Radboud University Medical Center Amalia Children’s Hospital Nijmegen the Netherlands; ^3^ Department of Vascular and Transplant Surgery Radboud University Medical Center Nijmegen the Netherlands; ^4^ Department of Intensive Care Medicine Radboud University Medical Center Nijmegen the Netherlands

**Keywords:** child, cristalloid solutions, critical care, education, equipment, fluids, general anesthesia, invasive, kidney transplantation, monitors, PICU, renal, young age

## Abstract

Living‐donor kidney transplantation is the first choice therapy for children with end‐stage renal disease and shows good long‐term outcome. Etiology of renal failure, co‐morbidities, and hemodynamic effects, due to donor‐recipient size mismatch, differs significantly from those in adult patients. Despite the complexities related to both patient and surgery, there is a lack of evidence‐based anesthesia guidelines for pediatric kidney transplantation. This educational review summarizes the pathophysiological changes to consider and suggests recommendations for perioperative anesthesia care, based on recent research papers.

**Reflective Questions**

What is the cardiovascular status of children with kidney failure when they present for surgery?

How to prepare for anesthesia care in the child with kidney failure?

How to monitor and support hemodynamics in pediatric kidney transplantation?

What is best practice anesthesia care for the child with kidney failure or a donor kidney?

## 1 INTRODUCTION

End‐stage renal disease (ESRD) in children is rare with an incidence of 5–15 per million depending on geographic location. The best treatment option is kidney transplantation, which delays end‐organ damage, enhances physical growth and improves quality of life. Advances in immunosuppressive therapy, surgical techniques and donor selection have improved both graft and patient survival over the last decades. Best outcomes are achieved with a pre‐emptive transplantation (before the initiation of dialysis) from a living donor and at a young recipient age (<5 years). The resulting one‐ and five‐year graft survival rates are then 99.5 and 94.9 percent, respectively.^1^

Because of the low incidence of kidney transplantation in children and the complexities related to patient and surgery, care tends to be centralized in designated centers. Moreover, only 20% of pediatric kidney transplantations occur in recipients under the age of five, making it a rare procedure for the pediatric anesthesiologist to encounter. Notwithstanding, there is a clear lack of evidence‐based guidelines resulting in diverse perioperative approaches.^2^, ^3^

The purpose of this review of recent literature is to describe the pathophysiological changes occurring in children with ESRD. In addition, we aim to provide recommendations and potential guidelines for anesthesia care in children undergoing either kidney transplantation or other surgical procedures in the presence of a donor kidney.

## 2 EPIDEMIOLOGY

Children account for less than 2% of all ESRD patients and about 5% of all kidney transplantations in Europe and North America. Kidney disease prevalence, etiology, and registration differ worldwide, so comparison between regions is difficult.

The etiology of ESRD in children, and therefore, the pathophysiological changes to consider, differ significantly from those in adults. In young children, common causes are congenital disorders such as dysplastic kidneys and obstructive uropathy. In older children, glomerulopathies such as focal segmental glomerulosclerosis are more prevalent.

Renal replacement therapies include hemodialysis, peritoneal dialysis, and kidney transplantation from a deceased or living donor. Timing and type of renal replacement therapy vary between countries, depending on cultural and economic factors. For example, in North America and Europe, transplantation is the ultimate renal replacement therapy. In some European countries, up to 50% of transplantations are performed using living donors, and in several countries, children are given priority on the waiting list. In contrast, Middle East and African countries have the lowest organ donation rates of any source.^4^ Most donor kidneys come from adult donors, but kidneys of small deceased pediatric donors (<20 kg) can also be transplanted. These small kidneys are often transplanted “en bloc”: two kidneys along with a part of the donor aorta and vena cava. Having the potential to grow along with a growing recipient, small pediatric kidneys show a good long‐term outcome when transplanted in older children or adult recipients.^5^ However, in small pediatric recipients, this procedure shows a high incidence of vascular complications. Therefore, adult donor kidneys are preferred for small recipients (<20kg).

## 3 HEMODYNAMIC PHYSIOLOGY

### 3.1 Cardiovascular changes in children with end‐stage renal disease

The pathophysiology of kidney disease affects various components of the hemodynamic system, causing hypertension and structural cardiovascular changes even at a young age.

Sodium homeostasis and blood pressure regulation are closely related through the pressure natriuresis system. In a healthy state, an increased blood pressure at the glomerulus results in decreased sodium reabsorption in the tubules, causing natriuresis and a reduction in circulating volume and blood pressure.^6^ In renal disease, this mechanism is impaired, which results in an increased circulating volume and thus increased myocardial preload and stroke volume. Furthermore, the left ventricular afterload is increased due to a chronically activated sympathetic nervous system (SNS). In the normal state, close interaction between the SNS and the kidneys maintains blood pressure and glomerular filtration rate (GFR). However, in diseased kidneys, the reduced GFR stimulates the renin‐angiotensin system and the afferent sympathetic output to the SNS.^7^ The increased SNS activity then results not only in hypertension, but also reduced heart rate variability and impaired baroreflex sensitivity.^8^ Additionally, uremia and electrolyte imbalances cause endothelial dysfunction and vascular calcifications. This vasculopathy causes increased arterial stiffness and therefore reduced compliance of the vascular tree, further augmenting the cardiac afterload.^9^ Consequently, both changes in preload and afterload increase the myocardial workload. Indeed, the majority of pediatric patients with renal failure show myocardial strain and left ventricular hypertrophy on echocardiography. Although systolic function is usually not impaired, diastolic dysfunction is often found. This puts children with ESRD at an increased risk for cardiovascular complications. Early transplantation has been shown to prevent impairment of baroreflex function and improve left ventricular function in children with ESRD and myocardial hypertrophy. Kidney transplantation in an early stage of kidney failure may therefore slow down cardiovascular pathophysiological changes.^10^

### 3.2 Hemodynamic physiology of the donor kidney

**FIGURE 1:**
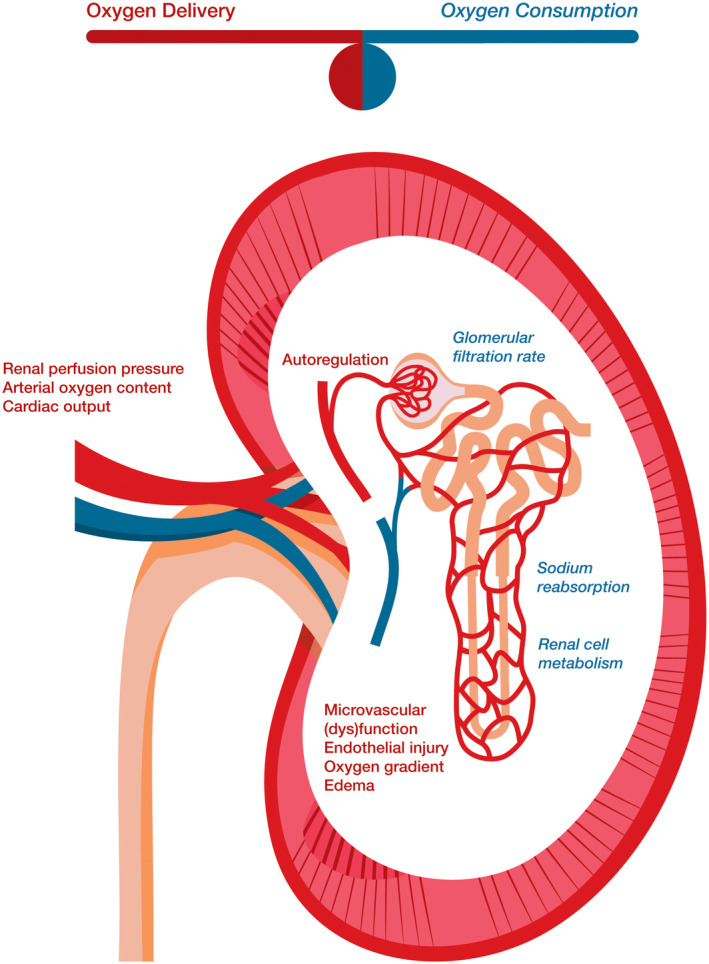
Factors contributing to kidney oxygen delivery and oxygen consumption in kidney transplantation. Oxygen delivery to the kidney is the product of renal blood flow and arterial oxygen content. Arterial oxygen content depends on hemoglobin concentration and arterial oxygen saturation. Renal blood flow is the resultant form cardiac output, intravascular volume, renal artery patency and autoregulation of renal perfusion pressure. When intact, intrarenal autoregulatory mechanisms regulate blood flow and pressure at the glomerulus, within a defined range of arterial blood pressure. Microvascular function, oxygen diffusion gradient and tissue properties play important roles in medullary tissue oxygenation. After kidney ischemia and reperfusion, oxygenation may be hampered due to edema and endothelial damage. Oxygen consumption is mainly determined by basic renal cell metabolism, glomerular filtration rate and sodium reabsorption. The medulla's microvascular structure and high oxygen consumption rate make this region more prone for a disbalance between oxygen delivery and oxygen consumption. Therefore, it is more affected by hypotension and hypoxia compared with the renal cortex

Ischemia and subsequent reperfusion render the donor kidney particularly vulnerable to tissue hypoxia and acute kidney injury (AKI). The challenge in kidney transplantation is to minimize kidney tissue hypoxia by balancing oxygen delivery and oxygen consumption (Figure 1).

At donor nephrectomy, oxygen delivery ceases completely. This is followed by a short warm ischemia period after which the donor kidney is rapidly cooled to minimize oxygen consumption and cell disintegration. This cold ischemia period enables storage of the donor kidney until transplantation. When the vascular anastomosis in the recipient starts, the kidney tissue will gradually warm to body temperature. Cell metabolism will subsequently start up and the warm ischemia period commences. This period ends with unclamping of the vessels after which renal blood flow is restored. Nonetheless, a mismatch between oxygen supply and demand, started in the warm ischemia period, is continued after reperfusion. Supplying oxygen after an ischemic period causes ischemia‐reperfusion injury resulting in endothelial damage and reduced autoregulation. This compromises microvascular perfusion and oxygen supply, particularly affecting the donor kidney's medulla with its easily obstructed narrow vasculature. Moreover, cellular damage induced tissue edema increases the oxygen diffusion distance between blood and renal cells, further impairing tissue oxygenation. Simultaneously, the restored renal blood flow triggers glomerular filtration and tubular sodium reabsorption, thus increasing kidney oxygen consumption and entailing a further mismatch between oxygen supply and demand. If the oxidative stress persists, interstitial fibrosis and partial loss of kidney function may occur. Kidneys from deceased donors, long ischemia times (>24 h) and increased donor age are known risk factors for this injury to occur, and are related to an increased incidence of acute tubular necrosis and delayed graft function (DGF).^11^, ^12^

Hypoxia had been shown to blunt renal autoregulation. Therefore, shortly after reperfusion, donor kidney perfusion pressure is sensitive to changes in systemic blood pressure and flow.^13^

## 4 PREOPERATIVE ASSESSMENT

An extensive preoperative assessment is of vital importance to acquire a clear picture of potential metabolic changes and end‐organ damage. In general, children with ESRD are frail, acidotic, anemic and hypertensive. However, with high‐quality supportive care, for example, optimal nutrition, fluid restriction, and prevention of acidosis, children may remain in a relatively good condition. In contrast, unsuccessful care may lead to malnutrition, neurological damage, severe electrolyte imbalances, bones and teeth deformities, hypervolemia, and congestive heart failure.

Table 1 summarizes the impact of renal failure on the functioning of various types of organs. Consequences for anesthesia are discussed in detail below.

**TABLE 1:** Preoperative assessment in pediatric kidney transplantation: tests and considerations

Organ system	Encountered problems	Preoperative tests	Preoperative preparation
Pulmonary function	Pulmonary edema Lung fibrosis Obstructive and/or restrictive lung function	Lung function tests SpO2 on room air	Perioperative bronchodilator therapy when indicated
Cardiac function	Hypertension Left ventricular hypertrophy, diastolic dysfunction, structural lesions Pericardial effusion	Blood pressure Cardiac ultrasound	Antihypertensive medication usually stopped on day before surgery, to prevent intra‐operative hypotension
Neurologic development	Cognitive impairment Uremic neurotoxicity		
Electrolytes	Hyperphosphatemia Hypocalcemia Hypomagnesemia Hyperkalemia Acidemia	Serum electrolyte analysis Blood gas analysis	Diet, Cation exchangers, Phosphate binders, Calcium, Magnesium supplements; all stopped on day of surgery Sodium bicarbonate; continued until surgery
Hemostasis	Uremic thrombopathy Anemia	Full blood count Blood Type and Crossmatching Coagulation tests	Pre‐order blood for transfusion when indicated Consider possible bleeding tendency
Fluids and feeding	Residual diuresis or anuria Hemo‐ or peritoneal dialysis Fluid restriction Malnutrition, feeding difficulties Delayed gastric emptying	Glucose (in)tolerance Signs of fluid overload	Gastric tube for feeding and medication Fluid restriction Glucose‐containing fluids when at risk for hypoglycemia Adjust fluid withdrawal by dialysis when indicated
General evaluation	Urologic impairment, risk of postrenal failure Vascular patency Immunologic status	Full urology assessment Imaging of abdominal organs and vasculature Serology for past infections	Voiding training Immunization
Hormones	Reduced erythropoietin Hyperparathyroidism Renal osteodystrophy; brittle teeth and bones Growth hormone deficiency	Hemoglobin test Serum calcium and phosphate	Erythropoietin therapy Growth hormone therapy in growth retardation Dental care
Nephrological disease	Nephrotic Syndrome Glomerulonephritis Syndrome associated co‐morbidities	Fluid and protein status Hypotension Hypertension	Nephrectomy prior to transplantation in active nephrotic syndrome Adequate fluid status and electrolyte control
Urologic disease	Obstructive uropathies Renal agenesis or dysplasia	Anuria in utero: lung hypoplasia Congenital cardiac disease	Urologic work up Cardiologic work up
Mental wellbeing	Perioperative anxiety		Consider (non)pharmacological therapy

### 4.1 Pulmonary function

Fluid overload and leakage of alveolar membranes can cause pulmonary edema and obstructive lung function. Uremia, anemia, and malnutrition also play a role in this pathogenesis. If the edema persists, interstitial fibrosis develops, causing a restrictive lung function. Therefore, lung function tests in children with ESRD may show a restrictive, obstructive or mixed pattern. A reduced oxygen reserve may be present, even when clinical signs are not manifest.^14^

### 4.2 Cardiovascular function

Cardiac changes and increased SNS activity are associated with a high incidence of hypertension. A preoperative electrocardiogram and echocardiography are required to assess the cardiovascular state and myocardial performance. Hypertension is often treated with angiotensin‐converting enzyme inhibitors or angiotensin receptor blockers. These drugs are the therapy of first choice, as they have been shown to slow down progression of chronic kidney disease in children.^15^ If necessary, a calcium antagonist is added. Antihypertensive drugs are usually stopped on the day of surgery, as the combination with the vasodilatory effects of anesthetics may cause refractory hypotension.

### 4.3 Bone mineralization

Reduced renal excretion of phosphates causes hyperphosphatemia, whereas hypocalcemia results from an impaired renal vitamin D production and a decreased intestinal calcium absorption. Both hyperphosphatemia and hypocalcemia cause deficiencies in mineralization and trigger the secretion of parathyroid hormone. This hormone stimulates calcium resorption form bones leading to renal osteodystrophy. Metabolic acidosis accelerates this process. Therefore, children with renal failure may present with brittle bones and teeth.^16^

### 4.4 Co‐morbidities

Specific co‐morbidities can be present, depending on the type of renal disease.

Severe types of obstructive uropathies and kidney dysplasia can cause reduced urine formation in utero, resulting in oligohydramnios and abnormal lung development. In these patients, reduced lung capacity may be present and should be assessed with lung function testing.

Kidney dysplasia is associated with congenital cardiac diseases, most frequently being a septal defect.

Nephrotic syndrome is characterized by glomerular leakage of proteins, edema, intravascular hypovolemia and, frequently, a difficult to manage hypotension. In active nephrotic syndrome with refractory hypoalbuminemia, a nephrectomy of the native kidneys and temporary dialysis prior to transplantation is recommended. A bilateral nephrectomy can result in persistent and difficult to treat hypotension.

Glomerulonephritis often leads to a rapid decline in kidney function and difficult to manage hypertension. As criteria for dialysis might be quickly reached, a pre‐emptive transplantation is therefore often not feasible.

Renal disease can have a variety of genetic causes leading to syndromes of morbidities. The associated morbidities of other organs may have additional consequences for anesthesia. Examples are Prune‐Belly, Alport, Denys‐Drash and Pierson syndrome.

### 4.5 Timing of transplantation

The timing of transplantation depends on progression of disease, success of conservative therapy and the availability of a suitable donor. A pre‐emptive living donor kidney transplantation can be prepared when optimal supportive care is successful and enables the child to grow. Physical growth is favorable, as a larger abdominal space and vasculature reduce the risk of vascular complications. Moreover, the donor‐recipient size mismatch related hemodynamic consequences are probably less pronounced in larger recipients. When pre‐emptive transplantation is not feasible, dialysis is started as a bridge to transplantation. Indications to start dialysis are fluid overload leading to congestion, untreatable hyperkalemia, excessive uremia, and cessation of growth and development due to lack of energy. Peritoneal dialysis is preferred, as it can be practiced at home and preserves veins and arteries. However, it bears the risk of peritonitis, sometimes necessitating a (temporary) switch to hemodialysis.

## 5 INTRAOPERATIVE CARE

Kidney transplantation is a combined vascular and urologic surgical procedure. The vascular anastomoses are commonly made to the iliac vein and iliac artery, preferably using the retroperitoneal approach. In small recipients (<20 kg), the donor kidney is frequently placed intraabdominal whereby the vascular anastomoses are made to the descending aorta and inferior vena cava. After reperfusion and apparent adequate perfusion of the graft, the ureter is anastomosed to the bladder.

Table 2 summarizes anesthesia management in pediatric kidney transplantation. Specific challenges are discussed in detail in the following paragraphs.

**TABLE 2:** Anesthesia care during pediatric kidney transplantation: checklist of considerations and advice

	Considerations & advice	Monitoring
Airway	Adjust tube and catheter sizes to patient's size, not to age	
Breathing	Lung protective ventilation (TV 6–8 ml/kg, PEEP 5–10 cmH_2_O) A reduced oxygen reserve might be present	Airway pressures Pulse oximetry Capnometry
Circulation	Reduced vascular compliance Left ventricle hypertrophy Diastolic dysfunction Anesthesia induced hypotension Set target arterial blood pressure, add vasopressors if fluid loading is insufficient	Intra‐arterial blood pressure Cardiac output and/or fluid responsiveness Central venous catheter
Anesthesia & analgesia	No clear advantages of one anesthetic over another; choose best cardiovascular profile No succinylcholine in hyperkalemia Preference for hepatic clearance or inactive metabolites if delayed graft function is anticipated	Neuromuscular monitoring
Electrolytes	Consider sodium bicarbonate solution as part of fluid therapy Treat hyperkalemia with calcium gluconate and/or glucose/insulin Prevent hyponatremia and brisk osmolality shifts	Regular checks of serum electrolyte and acid base status, for example, one hourly
Fluids	Reduce basic fluids in patient with anuria Beware of hypoglycemia when patient is on continuous feeding Fluid loading before graft reperfusion with isotonic & balanced solutions and mannitol Withhold fluid loading when signs of fluid overload appear: increased central venous pressure or pulmonary edema Compensate fluid losses; beware of poly‐uric phase in first hours after transplantation	Regular glucose control Fluid responsiveness Central venous pressure Urine output after reperfusion
Blood	Anemia; lower threshold to transfusion	Full blood count
Medication	Antibiotic prophylaxis; dose adjusted to body surface area Immune suppressive medication Thrombosis prophylaxis when indicated No rationale for diuretics or dopamine Mannitol; dose adjusted to body surface area Consider giving calcium gluconate at reperfusion to counteract hypotension and hyperkalemia	

### 5.1 Anesthesia technique

Kidney transplantation requires general anesthesia, endotracheal intubation and controlled ventilation. There is no specific preference for the choice of sedatives or opioids, although sevoflurane might show a relatively better hemodynamic profile compared with other inhalational anesthetics.^17^ After its introduction, there have been concerns about the nephrotoxicity of sevoflurane metabolites (Compound A) in rats. Although there are no specific data in children with renal failure or a kidney transplantation, many studies have failed to show clinical significant effects of sevoflurane on renal function in humans. Therefore, contemporary, it is considered safe in pediatric clinical practice.^18^

The use of medium to long‐acting neuromuscular blocking agents is preferably guided by neuromuscular monitoring to prevent inadvertent residual curarization. Succinylcholine is not advised for its risk to provoke hyperkalemia and dysrhythmias. Controlled deep neuromuscular blockade during the anastomosing phase of the transplantation can be reached using repeated doses of atracurium or rocuronium. Rocuronium has a higher risk of postoperative residual neuromuscular blockade, but reversal is rapidly achieved with sugammadex. The sugammadex‐rocuronium complex is excreted by glomerular filtration and its clearance is significantly reduced in renal failure. However, it is a stable complex and the occurrence of recurarization appears to be unlikely in patients with ESRD. Case reports in children and adult patients with renal failure did not reveal side effects of the use of sugammadex and suggest a good safety profile.^19^, ^20^ Moreover, in successful kidney transplantation glomerular filtration is quickly restored with unhampered filtration and elimination of the complex.

### 5.2 Mechanical ventilation strategy

Regarding the prevalence of reduced lung function, it seems reasonable to practice lung protective mechanical ventilation in children with ESRD. Although studies investigating the effect of pediatric mechanical ventilation practices on outcome are scarce, the Paediatric Mechanical Ventilation Consensus recommends the following strategy in critical ill children: 1) A tidal volume of 5–8 ml kg^−1^ with lower volumes in patients with lung hypoplasia. 2) Standard low PEEP levels (3–5 cm H_2_O) in noninjured lungs to prevent alveolar collapse; in progressive lung injury higher PEEP levels may be needed. 3) A plateau pressure <29–32 cm H_2_O for injured lungs, although this may be difficult to measure on most anesthesia ventilators.^21^

### 5.3 Hemodynamic monitoring

A crucially important hemodynamic goal in kidney transplantation is optimal graft perfusion, because inadequate perfusion and oxygenation increase the risk of postoperative acute tubular necrosis and DGF. Since microvascular blood flow cannot be monitored, nor directly influenced, hemodynamic monitoring focusses on arterial blood pressure, fluid status and systemic blood flow.

It could be argued to aim at a post‐transplantation mean arterial pressure (MAP) above 70 mmHg in pediatric recipients receiving an adult donor kidney. After all, in adults, autoregulatory mechanisms secure a stable renal blood supply in the MAP range of 60–80 mmHg.^22^ Furthermore, a post‐transplantation MAP below 70 mmHg in adult kidney transplant recipients appeared to be related to a higher incidence of DGF.^23^ However, the administration of excessive fluids or vasopressors to reach this target in young children might do more harm than a slightly lower MAP. Therefore, the target MAP might be adjusted depending on the donor's baseline MAP. Also, after release of the vascular clamps, the surgeon will visually judge whether the perfusion of the donor kidney is adequate or not. The MAP, at which adequate perfusion is reached, could be used as target in the postoperative period and might well differ from the “standard” target of 70 mmHg.

Besides a suitable MAP, adequate fluid status and blood flow are required. Clearly, both hypovolemia and fluid overload should be avoided. Hypovolemia causes inadequate tissue perfusion and oxygen delivery, whereas fluid overload causes tissue edema and reduced oxygen diffusion.^22^ Traditionally, a high target CVP was advocated to guide fluid therapy during pediatric kidney transplantation. CVP is, however, an unreliable indicator of fluid status, performs poorly in predicting fluid responsiveness, and is not related to the incidence of DGF in adult kidney transplantations.^23^, ^24^ Moreover, a high CVP is associated with an increased mortality in critical ill children.^25^ Furthermore, analyses of anesthesia management practices in pediatric kidney transplantation suggest a relationship between CVP guided fluid therapy, fluid overload, postoperative pulmonary edema and prolonged ventilator support.^26^, ^27^, ^28^

The key question in fluid therapy is whether extra fluids will increase the patient's cardiac output; in other words, “Is the patient fluid responsive?” Monitoring intravascular volume status or predicting fluid responsiveness is, however, difficult, especially in children. The Surviving Sepsis Campaign for children advises fluid administration to be guided by advanced hemodynamic monitoring and to be discontinued if signs of fluid overload develop. This hemodynamic monitor should preferably predict patient's fluid responsiveness, or otherwise at least track changes in cardiac output, systemic vascular resistance or mixed venous oxygenation.^29^ Fluid responsiveness is reliably predicted by stroke volume variation or pulse pressure variation in adults, but not in children. In children, respiratory variations in aortic blood flow peak velocity and esophageal Doppler peak velocity of the descending aorta have been shown to predict fluid responsiveness.^30^, ^31^

Cardiac output (CO) monitors are useful to assess systemic flow or guide fluid administration, but only few are suitable and reliable in children. The transpulmonary thermodilution CO‐monitor is accurate and has been validated in children, but needs an intravascular catheter in a large, mostly femoral, artery. A less invasive alternative is the trans‐esophageal Doppler monitor. Despite its limitations in measuring absolute values of CO, it is accurate in tracking flow changes and therefore useful to evaluate the effect of administered intravenous fluids.^32^ Adult kidney transplant patients received less fluids when fluid therapy was guided by esophageal Doppler compared with CVP monitoring, without differences in renal outcome.^33^ In children, only one study analyzed the effect of CO‐guided hemodynamic management on fluid therapy in pediatric kidney transplantations suggesting a similar beneficial effect.^34^

### 5.4 Hemodynamic therapy

Hemodynamic goals can be reached using a balanced mix of fluids and vasoactive drugs.

Fluid administration should cover basic metabolic needs, fluid load to prepare for reperfusion of the donor kidney and compensation of urine output. Maintenance fluids should cover insensible loss, urine output and caloric needs. For basic fluid need, approximately 50% of the standard 4–2–1 rule can be used in the anuric patient. Caloric needs are covered by adding glucose to the maintenance fluids. Higher glucose concentrations may be needed in children at risk for perioperative hypoglycemia, for example, children on parenteral or continuous enteral feeding. However, large amounts of hypotonic solutions should be avoided as they carry the risk of hyponatremia and subsequent brain swelling. Particularly in pediatric transplants, hypotonic solutions in conjunction with quickly resolving hyperuricemia can aggravate the osmolality disequilibrium between serum and cerebral cells leading to cerebral edema.^35^ Mannitol, given shortly before reperfusion of the graft, might reduce this risk. In addition, mannitol forces osmotic diuresis and is thought to serve as a free radical scavenger, thereby mitigating ischemic reperfusion damage and decreasing the incidence of acute renal failure after transplantation.^36^ Therefore, mannitol is included in most kidney transplantation protocols.

Before unclamping, anticipating on the concurrent hypotension caused by the release of waste products from the ischemic donor kidney, it is advised to give fluid loading. Balanced crystalloids like lactated Ringer's solution are preferred for fluid loading, because large volumes of normal saline can cause hyperchloremic acidosis.^37^ Although lactated Ringer's solution contains potassium, its use is not associated with hyperkalemia during kidney transplantation. Because the concentration of potassium in the lactated Ringer's is similar to that in the human serum, the volume expansion will not alter the concentration of potassium in the patient. Moreover, fluid loading with normal saline shows a higher risk of causing hyperkalemia as its induced acidemia might force potassium out of the cells in exchange for the excess serum hydrogen ions.^38^

Sodium bicarbonate solutions can be part of the fluid loading when metabolic acidosis, caused by renal bicarbonate loss, is present. Because large amounts of crystalloids may cause tissue edema, it could be argued to partly replace them by colloids. However, hydroxyethyl starch and gelatin have been shown to increase the risk of mortality, coagulopathy and acute kidney injury in adults with sepsis or septic shock. Also, the Surviving Sepsis Campaign for Children recommends against their use in septic pediatric patients. Therefore, synthetic colloids are not recommended in kidney transplantation. Albumin is a reasonable alternative, but it bears the, albeit very low, risk of blood‐borne infections and it is more expensive.^29^

Vasopressors, like norepinephrine, can be administered when the patient is not fluid responsive and target MAP is not yet reached. Norepinephrine counterbalances the anesthesia induced vasodilation. Also, after reperfusion of the donor kidney, norepinephrine can prevent the hypotension caused by the sudden decrease in systemic vascular resistance. Postreperfusion hypotension is particularly seen in small recipients with an adult donor and in recipients receiving a postmortem donor kidney. In the latter, the cold ischemia time of the graft causes increased amounts of vasodilating cytokines to be released into the circulation. The fear for norepinephrine's induced vasoconstriction to impair renal blood flow appears unfounded. On the contrary, in hypotensive, vasodilated patients with acute kidney injury, norepinephrine has been shown to restore blood pressure within autoregulatory values and improve renal blood flow.^39^ Inotropes are usually not needed to increase CO, as most pediatric recipients exhibit good cardiac systolic function. Low‐dose dopamine, mainly used for its putative reno‐protective effects, has been shown to be obsolete in kidney transplantation and bears the risk of beta‐mimetic induced tachycardia.^40^

A suggested algorithm for hemodynamic therapy in pediatric kidney transplantation is shown in Figure 2.34

**FIGURE 2:**
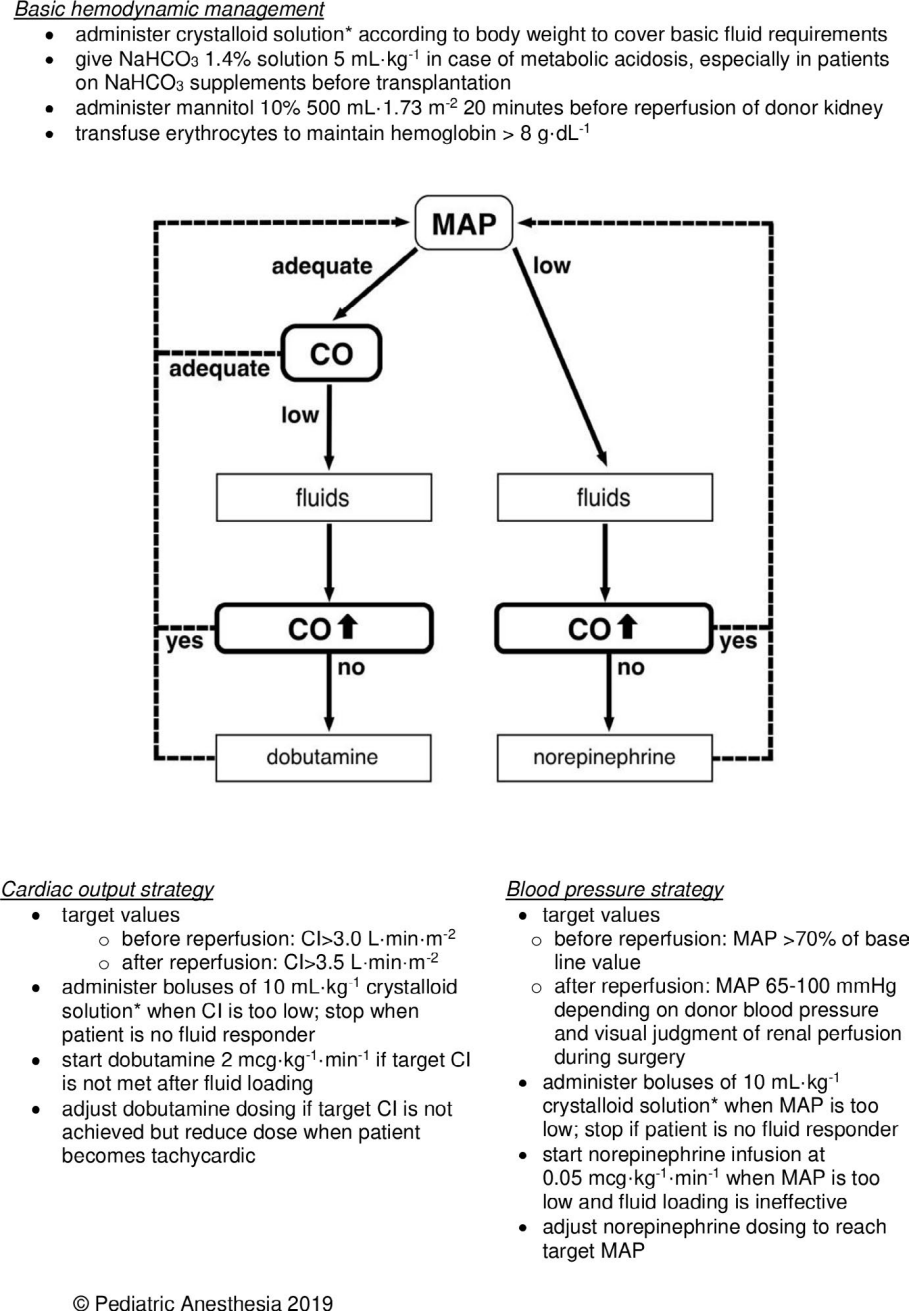
Algorithm of perioperative hemodynamic therapy in pediatric kidney transplantation guided by blood pressure and cardiac output measurements. NaHCO_3_ = sodium bicarbonate; MAP = Mean Arterial Pressure; CO = cardiac output; CI = Cardiac Index. Fluid responsiveness is defined as an increase in CO (or stroke volume) of >10%. ^*^Consider using balanced solution (like lactated Ringers’ solution) to prevent hyperchloremic acidosis

### 5.5 KIDNEY TRANSPLANTATION IN VERY YOUNG CHILDREN (AGE <5 YEARS)

Young kidney transplant recipients show a lower risk of acute rejection, which might result from their “naive” immunological status. In addition, immunologically compatible parents or other adult relatives often present themselves as living donors. The significant donor‐recipient size mismatch, however, implicates surgical and anesthetic challenges.^41^

First, vascular caliber size mismatch can result in compromised arterial blood flow and venous congestion. This is prevented by anastomosing the renal vasculature to the descending aorta and inferior vena cava.

Second, a relatively large adult kidney in a small child has hemodynamic consequences. Considering a blood flow to an adult kidney of approximately 500 ml min^−1^ and a CO of around 2000 ml min^−1^ for a two‐year‐old child, the recipient's CO has to increase 25% to meet the flow demands of the donor kidney. Therefore, optimal perioperative supportive hemodynamic therapy is required to prevent hypoperfusion and early ischemia of the graft. We would recommend to guide this therapy by advanced hemodynamic monitoring to prevent both hypovolemia and fluid overload.^34^

Still, despite optimal support, a reduction in graft blood flow compared with pretransplant flow seems inevitable in the weeks and months after transplantation. Initially, creatinine clearance increases sharply after transplantation, representing the donor kidney's relative overcapacity. But it declines in the first few months, probably due to adaptation to the recipient's CO and subsequent reduced donor kidney perfusion.^42^ This is illustrated by protocol graft biopsies in young recipients (body surface area <0.75 m^2^) showing nonimmunological damage suggestive for hypoperfusion. Also, the smallest recipients are not able to increase their functional renal capacity (eGFR) when they grow older and show more reduction in donor kidney size compared with their older counterparts.^43^, ^44^ This suggests that a persistently increased CO during the post‐transplant period might prevent loss of donor kidney tissue. However, this is challenging in young recipients as frequent infections and pre‐existing feeding difficulties increase the risk of hypovolemia. Also, early detection of donor kidney hypoperfusion is difficult, because the available imaging and biomarker monitors are of limited value.

## 6 POSTOPERATIVE CARE

Postoperative care should focus on optimal hemodynamic support and restoration of fluid and electrolyte imbalances. The pediatric intensive care unit is the location best suited for close monitoring and prompt treatment of early complications like acute tubular necrosis, DGF, electrolyte disorders, bleeding from anastomoses, arterial or venous thrombosis and pulmonary edema.

Ventilator support might be necessary when fluid overload, residual neuromuscular blockade or opioid activity is present. Hemodynamic support can be continued according to the same principles and considerations as advised in the Intraoperative Care section.

During the first 24 to 48 h, hourly monitoring and replacement of urine loss and electrolytes is mandatory. The sudden high glomerular filtration of urea bears the risk of an osmolality disequilibrium between serum and cerebral cells, especially in the smallest recipients. Additionally, large amounts of sodium, potassium, magnesium and phosphates are lost into a high urine output. Therefore, close monitoring and replacement of electrolytes is necessary to prevent brisk osmolality shifts and subsequent complications.^35^

A decrease in urine output might reveal early graft dysfunction due to rejection, relative hypovolemia or vascular complications. The latter can be confirmed or excluded by renal Doppler ultrasound. Advances in immunosuppressive therapy have made vascular thrombosis a more suspect cause of early graft failure than rejection. Risk factors for thrombosis are young recipient age (<5 years), young donor age (<5 years), long cold ischemia times and surgical complications. Whether the prophylactic use of anti‐coagulants, like heparin, prevents this complication is still a matter of debate.^45^

Postoperative pain therapy mainly consists of paracetamol and opioids. Morphine can be used without fear for accumulation of active metabolites if the graft functions well. A shift to opioids without active metabolites, like piritramide, can be preferred when DGF is suspected. Nonsteroidal anti‐inflammatory drugs are contraindicated as they compromise renal capillary blood flow. Epidural analgesia in pediatric kidney transplantation has been reported to have favorable outcome and relatively stable hemodynamics.^28^ However, its common practice is probably hampered by the fear for potential hemodynamic instability and epidural hematoma or abscess formation in patients with renal failure.

Late complications after kidney transplantation are hypertension, graft failure or rejection, infections and malignancies. Hypertension is seen in 50–80% of the recipients, although 75% of them did not have hypertension prior to transplantation. It is most frequently caused by the side effects of immunosuppressive medication, stenosis of the arterial anastomosis or graft rejection. Post‐transplant hypertension is related to a lower GFR at one‐year post‐transplantation and reduced graft survival rates.

Graft failure is related to several factors. First, the incidence is related to increased ischemia times, postmortem donors and older donor age. These factors are probably associated with more extensive ischemia‐reperfusion injury. Second, the immunosuppressive calcineurin inhibitors are nephrotoxic and sometimes dosing adjustments are required to prevent further kidney damage. Third, particularly juvenile recipients show reduced therapy adherence, causing a peak in graft failure rates between 17–25 years of age.^16^

A high infection rate is seen in the first‐year post‐transplantation. This occurs particularly in preschool recipients due to their immature immune system combined with the immunosuppressive therapy. Hospital admission is often required to guarantee oral intake and prevent hypovolemia and kidney damage.

Malignancies are a late complication of any organ transplantation due to the life‐long immunosuppressive therapy. In the North American (NAPRTCS) database 2.3% of pediatric kidney recipients developed a malignant disorder within three years after transplantation, lymphoproliferative disorders having the highest incidence.^1^

## 7 POST‐TRANSPLANT ASSESSMENT OF RENAL PERFUSION AND FUNCTION

Surveillance of post‐transplant kidney function is mandatory to detect early deterioration of kidney function, enabling therapy to start and prevent further renal impairment. Regular serum creatinine measurements are standard practice to calculate the estimated glomerular filtration rate (eGFR). However, creatinine is a late marker, its predictive value for graft loss is limited and it cannot differentiate between causes of graft injury, like rejection, hypovolemia or drug nephrotoxicity.^46^ Moreover, in the youngest recipients with an adult‐sized kidney and functional overcapacity, creatinine might only increase after significant renal tissue loss. Cystatin C is an alternative marker to assess eGFR, but has the same drawbacks as creatinine and shows more ethnic differences. More recent biomarkers are urinary and plasma chemokines released with renal cell disintegration. KIM1, NGAL and IL18 show promising results as early detection markers of renal tissue damage, but have mainly been studied in adults and CKD. Their value in pediatric kidney transplantation needs further investigation. Therefore, kidney biopsy is still the diagnostic gold standard when function deteriorates.^47^

Renal imaging can monitor perfusion and oxygenation of the graft more directly. Clearly, this requires nonradiation imaging techniques without the use of nephrotoxic contrast dye. Regular renal ultrasound imaging during follow‐up can assess urological function, provide color‐doppler images of regional flow and measure resistance to arterial blood flow by calculating resistive indices. A resistive index above 0.8 has been associated with poor graft function and survival. It is, however, a nonspecific marker which is influenced by multiple factors and cannot differentiate between causes of dysfunction. Small pediatric recipients with an adult‐sized donor kidney show higher resistive indices and a larger reduction in donor kidney volume compared with larger recipients.^48^ This possibly represents adjustment of the adult‐sized donor kidney to the lower renal artery flow in these recipients.

New techniques in Magnetic Resonance Imaging (MRI) show promising results in imaging renal perfusion and oxygenation in adult patients. Arterial Spin Labeling (ASL) MRI and Blood Oxygen Level‐Dependent (BOLD) MRI selectively label arterial blood water or deoxyhemoglobin, respectively, which makes imaging possible without the need for contrast dye.

Another noninvasive technique to measure donor kidney oxygenation is the near‐infrared spectroscopy (NIRS). The NIRS sensor is applied on the skin surfacing the organ of interest. The sensor emits light in the near‐infrared spectrum and detects its reflection after passing through the tissue. Because oxyhemoglobin and deoxyhemoglobin absorb red light at different wavelengths, the changes in the reflected light spectrum represent the ratio between the tissue's oxygenated and deoxygenated hemoglobin. In pediatric kidney transplants, oxygenation values appeared to correlate with resistive indices and systolic blood pressure, but not with eGFR. Further research is needed to assess its value as an early detector of post‐transplant donor kidney perfusion problems.^49^

## 8 ANESTHESIA FOR THE CHILD WITH KIDNEY DISEASE

Children with kidney disease or a donor kidney may need anesthesia for a variety of surgical procedures. This might be general surgery or procedures related to graft dysfunction, like kidney biopsy, placement of dialysis catheters or urological procedures. Anesthesia preparations should be done in close cooperation with the nephrologist and are comparable to those described in the Preoperative Assessment section and Table 1.

Even in kidney transplant recipients with a well‐functioning donor kidney, pathophysiological changes must be considered. End‐organ damage caused by the prior kidney disease might resolve, preserve or worsen after transplantation. For example, pulmonary edema probably resolves, but restrictive lung function due to lung fibrosis might persevere. The cardiovascular pathophysiology probably progresses, albeit in a slower fashion compared with continuation on dialysis. This mainly results from the side effects of the immunosuppressive therapy. Fortunately, the newer immunosuppressive regimens have reduced or withdrawn the cortico‐steroids thereby decreasing the incidence of diabetes, metabolic syndrome and hypertension. Additionally, the newer calcineurin inhibitors show a better cardiovascular profile. However, diastolic dysfunction, hypertension and left ventricular hypertrophy are still seen in most recipients. This implies a persistent increased risk of cardiovascular events in future life.^50^, ^51^

During anesthesia, surgical stress, hemodynamic changes and fluid shifts are potential risks for kidney hypoperfusion and postoperative reduced graft function. Therefore, fluid status and blood pressure should be cautiously monitored and optimized. Clearly, any medication with a nephrotoxic profile, like NSAID’s, nephrotoxic antibiotics or contrast dye, should be withheld in these patients.

Last but not least, the anesthesiologist should be aware of the psychological burden and anxiety that is experienced around surgery and anesthesia, both by patient and parents. They have to cope with a history of frequent hospital admissions and interventions and might fear the risk of graft function loss due to surgical or anesthesia procedures. Therefore, care must be taken to detect post‐traumatic stress reactions.^52^

## 9 SUMMARY

Pediatric kidney transplantations show good long‐term outcomes, provided that optimal medical care is delivered. Anesthesia care for pediatric kidney transplantation requires thorough understanding of the pathophysiological processes in the child with ESRD. Furthermore, perioperative care differs substantially from that in adults. Clinically important differences relate to the etiology of the kidney failure, co‐morbidities and hemodynamic effects associated with organ‐donor size mismatch. Optimal perioperative hemodynamic support is crucial to secure adequate perfusion and oxygenation of the donor kidney, particularly when significant hemodynamic changes are anticipated as in small recipients receiving an adult‐sized kidney. Advanced hemodynamic monitoring can be used to guide hemodynamic therapy in order to reach target arterial blood pressure and prevent both hypovolemia and fluid overload. CVP guided fluid administration is considered of limited value and bears the risk of fluid overload and related complications. Even with a well‐functioning donor kidney, the incidence of hypertension and subsequent cardiovascular disorders remains high. As a result, pediatric kidney transplant patients exhibit a life‐long increased risk for cardiovascular events.

## Data Availability

Data sharing is not applicable to this article as no new data were created or analyzed in this study.
